# Proximal tubular deletion of superoxide dismutase-2 reveals disparate effects on kidney function in diabetes

**DOI:** 10.1016/j.redox.2025.103601

**Published:** 2025-03-18

**Authors:** Inez A. Trambas, Lilliana Bowen, Vicki Thallas-Bonke, Matthew Snelson, Karly C. Sourris, Adrienne Laskowski, Michel Tauc, Isabelle Rubera, Guoping Zheng, David C.H. Harris, Phillip Kantharidis, Takahiko Shimizu, Mark E. Cooper, Sih Min Tan, Melinda T. Coughlan

**Affiliations:** a Department of Diabetes, School of Translational Medicine, Monash University, Melbourne, 3004, Victoria, Australia; b Laboratoire de Physiomédecine Moléculaire, Université Côte D'Azur, CNRS, LP2M, 7370, Nice Cedex 2, France; c Centre for Transplantation and Renal Research, Westmead Institute for Medical Research, University of Sydney, Sydney, NSW, 2145, Australia; d Department of Food and Reproductive Function Advanced Research, Juntendo University Graduate School of Medicine, Bunkyo-ku, Tokyo, 113-8421, Japan; e Baker Heart and Diabetes Institute, Melbourne, 3004, Victoria, Australia; f Drug Discovery Biology, Monash Institute of Pharmaceutical Science, Monash University Parkville Campus, 381 Royal Parade, Parkville, 3052, Victoria, Australia

**Keywords:** Diabetes, MnSOD, SOD2, Diabetic kidney disease, Oxidative stress, Proximal tubules

## Abstract

There is a large body of evidence implicating mitochondrial reactive oxygen species (ROS) overproduction and oxidative stress in the development of diabetic kidney disease and the deficiency of mitochondrial antioxidant systems in the kidney, such as manganese superoxide dismutase (MnSOD/SOD2) have been identified. The proximal tubules of the kidney are densely packed with mitochondria thereby providing energy via oxidative phosphorylation in order to drive active transport for proximal tubular reabsorption of solutes from the glomerular filtrate. We hypothesized that maintenance of MnSOD function in the proximal tubules would be critical to maintain kidney health in diabetes. Here, we induced targeted deletion of SOD2 in the proximal tubules of the kidney in *Ins2*^Akita^ diabetic mice (SOD^ptKO^ mice) and show that 20 weeks of SOD2 deletion leads to no major impairment of kidney function and structure, despite these mice displaying enhanced albuminuria and kidney lipid peroxidation (8-isoprostanes). Plasma cystatin C, which is a surrogate marker of glomerular filtration was not altered in SOD^ptKO^ diabetic mice and histological assessment of the kidney cortex revealed no change in kidney fibrosis. Thus, our findings suggest that deletion of SOD2 in the proximal tubular compartment of the kidney induces a more subtle phenotype than expected, shedding light on the involvement of SOD2 and the proximal tubular compartment in the pathogenesis of diabetic kidney disease.

## Highlights

• Excessive oxidative stress is a key pathogenic mechanism of diabetic kidney disease.
• Manganese superoxide dismutase protects cells from excessive oxygen radicals.
• Although proximal tubule deletion of MnSOD does not impair renal function in diabetes, it leads to increased albuminuria. This highlights the complexity of this enzyme system in the kidney.

## Graphical abstract

**Figure:**
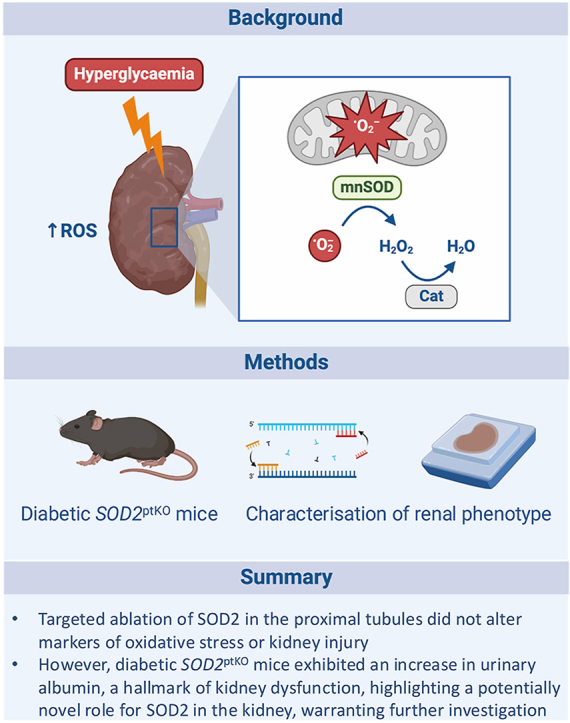

## 1 Introduction

Globally, more than 530 million people are living with diabetes mellitus [1]. Approximately 30–40 % of individuals with diabetes will eventually develop a kidney complication, referred to as diabetic kidney disease (DKD) [2]. DKD is characterised by persistent proteinuria, hypertension, and reduced glomerular filtration rate (GFR) [3]. While the thickening of the basement membrane of the glomerulus is the earliest histological change reported in DKD [4], changes in tubular structure and function, particularly of the proximal tubules, contribute significantly to disease progression [[5], [6], [7]]. Despite recent advances in the clinical management of DKD, a significant proportion of patients still progress to end-stage kidney disease and require kidney replacement strategies to avoid death. Thus, further advances in our understanding of DKD, leading to better treatments and biomarkers of DKD progression are urgently needed.

The kidneys are the second-highest consumers of molecular oxygen in the body after the myocardium and contain a high density of mitochondria, which are particularly abundant in proximal tubules [8]. Proximal tubular cells use oxygen to drive the process of active tubular reabsorption, returning sodium, glucose, ions, and other important metabolites back to the blood. During oxidative phosphorylation (OXPHOS) under physiologic conditions, there is minimal superoxide (O_2_^•^^−^) leakage from the respiratory chain, which is immediately scavenged by the intramitochondrial antioxidant enzyme, manganese superoxide dismutase (MnSOD, SOD2); however, damaged or dysfunctional mitochondria generate excessive superoxide, creating a state of redox imbalance. Excessive mitochondrial electron transport chain activity, causing an overproduction of reactive oxygen species (ROS), in particular O_2_^•^^−^, has been implicated as a key mechanism in the pathogenesis of diabetic kidney disease [[9], [10], [11], [12], [13], [14]].

Manganese superoxide dismutase (MnSOD/SOD2) catalyses the dismutation of superoxide anion radicals to hydrogen peroxide and molecular oxygen, dampening oxidative stress and regulating cellular oxygen [15]. MnSOD is considered critical for development and survival, with global knockout of *Sod2* in mice being neonatally lethal, with mice displaying dilated cardiomyopathy and metabolic acidosis [16]. MnSOD has been implicated in the pathogenesis of DKD through a single nucleotide polymorphism, V16A (Val [16]Ala) in Danish, Swedish and Finnish type 1 diabetes patients [17,18], suggesting that this MnSOD defect may increase the risk of developing DKD. Moreover, *SOD2* SNPs in individuals with kidney disease can act as an independent risk factor for the development of end-stage kidney disease [19].

Previous studies from our laboratory have shown a decline in MnSOD enzyme activity in the kidneys of streptozotocin-induced diabetic rats, four weeks after diabetes onset, which is evident prior to overt DKD, and persists throughout diabetes duration of 24 weeks [20]. We aimed to determine if deletion of *Sod2* the proximal tubules of the kidney: (i) is a primary cause of renal impairment, and (ii) can accelerate the progression of DKD, by generating a novel mouse model with a targeted knockout of *Sod2* within the proximal tubules.

## 2 Materials and methods

### 2.1 Generation of a mouse model with a targeted deletion of MnSOD in the proximal tubules of the kidney

All animal experiments were approved by the Alfred Research Alliance (ARA) Animal Ethics Committee (E/1485/2014/B) and performed in accordance with the research guidelines set out by the National Health and Medical Research Council of Australia and the ARRIVE guidelines. SOD2^flox/flox (fl/fl)^ mice (Dr Takahiko Shimizu, Chiba University, Japan) were rederived using IVF by Trans Genic Inc. (Chuo-ku Kobe, Japan). Upon importation to Australia, mice were backcrossed 11 generations to create SOD2^fl/fl^ mice on a C57BL/6J background. To generate the proximal tubular-specific SOD2 knockout (SOD^ptKO^) mice, *Sod2*^fl/fl^ mice were crossed with mice expressing Cre recombinase under the Sodium glucose cotransporter-2 (Sglt2) promoter (Sglt2-Cre), which directs Cre expression predominantly to the proximal tubular cells [21]. Sglt2-Cre mice were maintained on a C57BL/6J background. The gender matched *Sod2*^*flox/flox*^ littermate mice were used as control (designated *Sod2*^*Ctrl*^).

### 2.2 Isolation of tubules and verification of SOD2 deletion

Tubules were isolated from the renal cortex by using a differential sieving method. Briefly, glomerular and tubular fractions were obtained by mincing the renal cortex with a sterile scalpel and grinding the homogenate through a 100-μm disposable filter (BD Biosciences, San Jose, CA, USA) using a rubber syringe plunger. The saline-washed flow through material was passed through a 70-μm disposable filter. The glomeruli were collected on a 70-μm filter and the flow through material was collected to generate a tubular fraction. Genomic DNA was isolated from the renal cortex as well as from glomerular and tubular fractions of the renal cortex by using Maxwell® 16 DNA Purification Kits (Promega Corporation, Madison, USA) and used subsequently for genotype analysis by using a PCR method of GoTaq® DNA polymerase and gel electrophoresis from Promega. The probe and primer sequences are shown in Table 1.

**Table 1:** Primer sequences for confirmation of genotype.

Primer no	Sequence 5′-3′
P1	5′-CGAGGGGCATCTAGTGGAGAAG-3′
P2	5′-TTAGGGCTCAGGTTTGTCCAGAA-3′
P3	5′-CTAGTGAGATGGCTCAGC-3′
P3a	5′-GAGATGGCTCAGCGGTTAAA-3′
P3b	5′-CTAGTGAGATGGCTCAGCGGT-3′

### 2.3 MnSOD immunohistochemistry

Paraffin sections of mouse kidney (3 μm thick) were immunostained for SOD2 ((D9V9C) Rabbit mAb #13194, Cell Signalling Technology, Danvers, MA, USA). Endogenous peroxidases were blocked with 3 % hydrogen peroxide for 20 min at room temperature, washed, and subsequently incubated in 0.5 % skim milk powder for 30 min in a humidified chamber at room temperature to block excess binding. Sections were stained with the primary antibody (1:100) overnight at 4 °C. This was followed by incubation with biotinylated secondary antibody (1:500, Vector bio-anti rabbit Ab, Vector Laboratories, Newark, CA, USA) at room temperature for 10 min. Sections were then incubated with Vectastain ABC reagent (Vector Laboratories) for 30 min at room temperature. Peroxidase activity was identified by reaction with 3,3′-diaminobenzidine tetrahydrochloride (Sigma Aldrich, St Louis, MO, USA) for 2 min at room temperature. Sections were then oxidised with periodic-acid solution for 5 min at room temperature and subsequently stained with Schiff's reagent for 5 min at room temperature. Sections were examined under Upright Brightfield Nikon Ci microscope running NIS-Elements software (Nikon, Tokyo, Japan).

### 2.4 Experimental diabetes

*Ins2*^Akita^ (Akita) mice modelling a type 1 diabetic phenotype were used in this study. Akita mice harbour a spontaneous single nucleotide substitution in the *Ins2* gene [22]. The resultant abnormal folding of the insulin protein results in proteotoxicity to the pancreatic β-cells, and reduced production of mature insulin [23]. Homozygosity of the *Ins2* mutation is lethal, hence heterozygous mice are used. Akita mice on a C57BL/6 background reliably exhibit hallmarks of diabetic kidney disease including hyperglycaemia, albuminuria, and mesangial expansion [23]. The resultant *Sod2*^fl/fl^ Sglt2-Cre mice were crossed with heterozygous Akita mice to achieve *Sod2*^flox/flox^ Sglt2-Cre x Akita mice. Animals were housed in groups of 3–5 per cage in a temperature-controlled environment, under 12 h light/dark cycles with ad libitum access to food (standard chow; Speciality Feeds, Glen Forrest WA, Australia) and water. Nondiabetic and diabetic *Sod2*^Ctrl^ mice and *Sod2*^ptKO^ were followed for 20 weeks (n = 15 per group), with animals entering the study at 6 weeks of age. To ensure robustness, the mouse studies were conducted across eight cohorts, with a combination of genotypes included within each cohort to account for potential batch effects. At 20 weeks of the study, mice were housed individually in metabolic cages (Iffa Credo, L'Arbresele, France) for 24 h and urine was collected. Blood glucose and body weight were monitored weekly. Blood glucose was measured using a glucometer (Accutrend; Boehringer Manheim Biochemica, Manheim, Germany). Glycated haemoglobin (HbA1_c_) was determined using a Cobas Integra 400 Autoanalyzer (Roche Diagnostics Corporation, USA). After 20 weeks, animals received an overdose of sodium pentobarbital (160 mg/kg i.p.) and the kidneys were rapidly dissected, weighed, and snap-frozen or placed in 10 % neutral buffered formalin (v/v) for fixation before paraffin embedding.

### 2.5 Assessment of renal injury

Urinary albumin was determined by enzyme-linked immunosorbent assay (ELISA, Bethyl Laboratories Inc., TX, USA) [24]. Plasma Cystatin C (R&D Systems Inc., MN, USA) and urinary kidney injury molecule-1 (KIM-1, R&D Systems Inc, MN, USA) were measured by ELISA. For plasma Cystatin C, samples were diluted 1:1600, while KIM-1 samples were assayed either undiluted (mice with diabetes) or 1:8 (nondiabetic animals). Plasma glucose was measured using a glucose colorimetric assay kit (Cayman Chemical, Ann Arbor, MI, USA). For renal histology, kidney sections (3 μm) were stained with periodic-acid Schiff (PAS) [24]. The degree of sclerosis in each PAS-stained glomerulus was subjectively graded in a blinded manner, to obtain a glomerulosclerotic index, as previously described [25].

### 2.6 15-Isoprostane F2t concentration

Urinary 15-isoprostane excretion was measured by a competitive ELISA designed for mouse urine, as per the manufacturer's instructions (Oxford Biomedical Research Inc., Oxford, MI, USA). All samples were diluted 1:4 prior to the ELISA, and excretion was standardised to the 24-h urine volume.

### 2.7 Quantitative reverse transcription-polymerase chain reaction

RNA was isolated from the renal cortex (20–30 mg) using TRIzol Reagent (Life Technologies, NY, USA). DNA-free RNA was reverse transcribed into cDNA using the Superscript First Strand Synthesis System according to the manufacturer's specifications (Life Technologies BRL, Grand Island, NY). Real-time qPCR was performed using TaqMan assays as previously described [25]. The probe and primer sequences are shown in Table 2. Gene expression was normalised to 18S rRNA, and the relative fold change in expression was calculated using the comparative 2^−ΔΔCt^ method.

**Table 2:** Probe and primer sequences for qPCR.

Gene	Probe (5′-3′)	Forward primer (5′-3′)	Reverse primer (5′-3′)
Catalase (*Cat)*	P6-FAM CACTGACGTCCACCC	TTCAGAAGAAAGCGGTCAAGAAT	GATGCGGGCCCCATAGTC
Collagen I (*Col1a1)*	6- FAM ATCGACCCTAACCAAG	GACTGGAAGAGCGGAGAGTACTG	CCTTGATGGCGTCCAGGTT
Collagen IV (*Col4a1*):	P6-FAM CAGTGCCCTAACGGT	GGCGGTACACAGTCAGACCAT	GGAATAGCCGATCCACAGTGA
Copper-zinc superoxide dismutase (*Sod1*)	P6-FAM TGTGATCTCACTCTCAGGAG	GGACGGTGTGGCCAATGT	CGGCCAATGATGGAATGC
Fibronectin (*Fn1)*	P6-FAM CCCCGTCAGGCTTA	ACATGGCTTTAGGCGGACAA	ACATTCGGCAGGTATGGTCTTG
Glutathione peroxidase 1 (*Gpx-1*)	P6-FAM CGACCCCAAGTACATC	CCCCACTGCGCTCATGA	GGCACACCGGAGACCAAA
Manganese superoxide dismutase (*Sod2)*	P6-FAM CCTGAGCCCTAAGGG	GGGACATATTAATCACACCATTTTCTG	CCCAAAGTCACGCTTGATAGC
NAD(P)H dehydrogenase quinone 1 (*Nqo1)*	SYBER	CCAGCTGCTCAGCCAATCA	GCCATGGCTCCAGATGTTG
Transforming growth factor beta-induced (*Tgfbi*)	P6-FAM CAAAGATGGTGTCCCTC	GCCCCCCTGAATTCTGTGT	AGTCTTCATCTGGGCGTCGAT

### 2.8 Statistics

All statistical computations were performed using GraphPad Prism version 9.4.0 (GraphPad Software, San Diego, CA, USA). Outliers were removed using the ROUT method set at Q = 1 %. Experimental groups are shown as individual data points with a bar showing the standard error of the mean (SEM). The effect of genotype and disease status were tested using two-way analysis of variance (ANOVA), with Tukey's multiple comparison *post hoc* analysis. P values < 0.05 were considered to be statistically significant.

## 3 Results and discussion

### 3.1 Characterization of proximal tubular cell-specific SOD2 (*SOD2*^ptKO^) knockout mice

To elucidate the contribution of SOD2 to regulating oxidative stress in the mitochondria-abundant proximal tubules, we established a murine model of diabetes with a targeted SOD2 deletion. To generate the *Sod2*^ptKO^ mice, *Sod2* ^fl/fl^ mice were crossed with mice expressing Cre recombinase under the Sglt2 promoter (Sglt2-Cre). The resulting mice were crossed with *Ins2*^Akita^ mice to generate diabetic *Sod2*^ptKO^ mice (Fig. S1A) and mice were allocated to experimental groups as per Fig. S1B (n = 15 per group). Three mice died before the end of the study, due to diabetes-related illness (two diabetic *SOD2*^Ctrl^ mice and one *SOD2*^ptKO^ mouse).

To confirm generation of the *Sod2*^ptKO^ mouse, DNA was isolated from glomerular and tubular fractions of kidney cortex. Using the P1/P3 primer combination, a 2050 base pair (bp) band was observed in the presence of the floxed SOD2 allele (Fig. S2A), and a 401bp band was present in kidney cortex, tubules and glomeruli from *Sod2*^ptKO^ mice (Fig. S2B). The glomerular fraction is enriched for glomerular cells but also contains some contaminating proximal tubular cells, thus the 401bp knockout band is also present in this fraction.

To confirm proximal tubule-specific knockout of SOD2, a combination of immunohistochemistry and Periodic Acid Schiff (PAS) staining was performed. Proximal tubules were identified by a pink-stained brush border within the tubular lumen, while the absence of a brush border indicates distal tubules. A visible reduction of SOD2 immunoreactivity in the proximal tubules was observed in *Sod2*^ptKO^ mice (Fig. S2D, middle, closed arrows), indicating that protein expression of SOD2 was largely absent in proximal tubules in *Sod2*^ptKO^ mice, confirming the success of the targeted knockout.

**Fig. 1:**
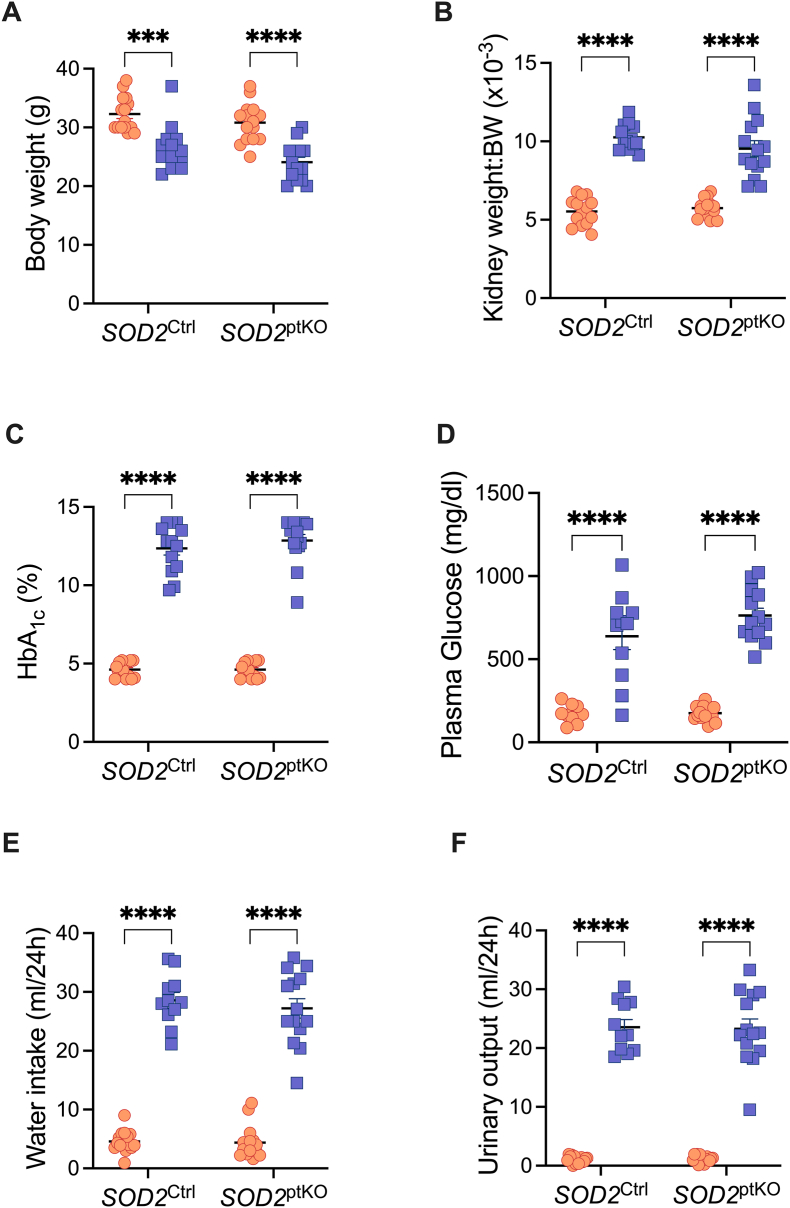
**Phenotypic and metabolic characteristics of mice at 26 weeks of age.** A) Body weight, n = 13 to 15 per group. B) Kidney weight:Body weight ratio, n = 13 to 15 per group. C) HbA_1C_, n = 13 to 15 per group. D) Plasma glucose n = 9 to 14 per group. E) Water intake, n = 12 to 15 per group. F) Urinary output (ml/24h), n = 12 to 15 per group. Dots and squares represent individual mice. Orange dots are non-diabetic mice. Blue squares are mice with diabetes (Akita). *P* values were determined by two-way ANOVA with Tukey's multiple comparison test. ∗∗∗*P* < 0.001, ∗∗∗∗*P* < 0.0001.

Non-diabetic SOD2^ptKO^ developed normally, with kidneys from knockout mice similar to SOD2^Ctrl^ mice at 25–26 weeks. Diabetic SOD2^Ctrl^ and diabetic SOD2^ptKO^ both displayed a significant decrease in body weight and increased relative kidney weight, accompanied by significantly increased HbA_1c_ and blood glucose (Fig. 1A–D), consistent with the *Ins2*^Akita^ model of diabetes [23]. Further, both diabetic groups displayed significantly increased water intake, in addition to increased urinary output (Fig. 1E–F) over 24 h. These data indicate that morphologically and metabolically, SOD2^ptKO^ mice did not differ significantly from SOD2^Ctrl^ mice.

### 3.2 SOD2 deletion in proximal tubules worsens diabetes-induced albuminuria but not renal structural injury

**Fig. 2:**
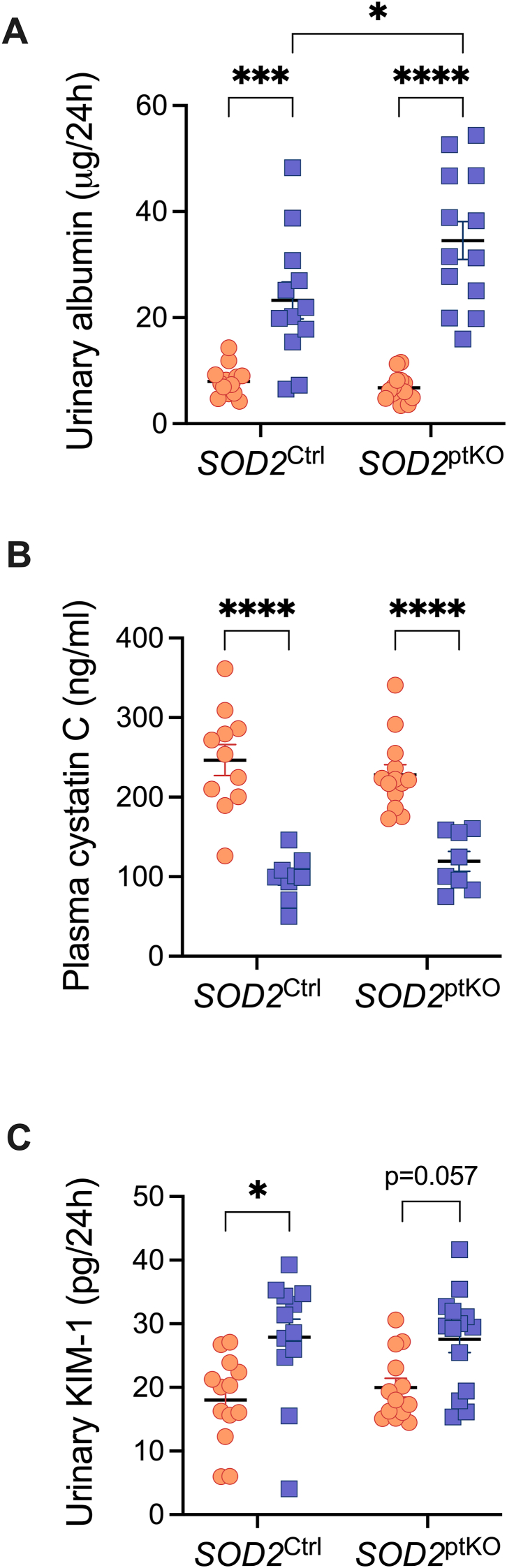
**Kidney injury.** A) Urinary albumin, n = 12 to 14 per group. B) Plasma cystatin C, n = 9 to 15 per group, and C) Urinary KIM-1, n = 13 to 14 per group. Dots and squares represent individual mice. Orange dots are non-diabetic mice. Blue squares are mice with diabetes (Akita). *P* values were determined by two-way ANOVA with Tukey's multiple comparison test. ∗*P* < 0.05, ∗∗∗*P* < 0.001, ∗∗∗∗*P* < 0.0001.

Next, we determined the renal function of the mice. A significant increase in urinary albumin excretion, a hallmark of DKD, was observed in both diabetic mice groups (Fig. 2A). Moreover, knockout of SOD2 in the proximal tubules worsened diabetes-induced albuminuria (Fig. 2A). In the setting of diabetes, both genotypes displayed renal hyperfiltration to a similar degree, as indicated by reduced plasma cystatin C [26] (Fig. 2B). Urinary KIM-1, a marker of proximal tubular injury [27], was significantly increased in SOD2^Ctrl^ mice with diabetes, however, this did not reach significance in SOD2^ptKO^ mice (Fig. 2C, p = 0.057).

**Fig. 3:**
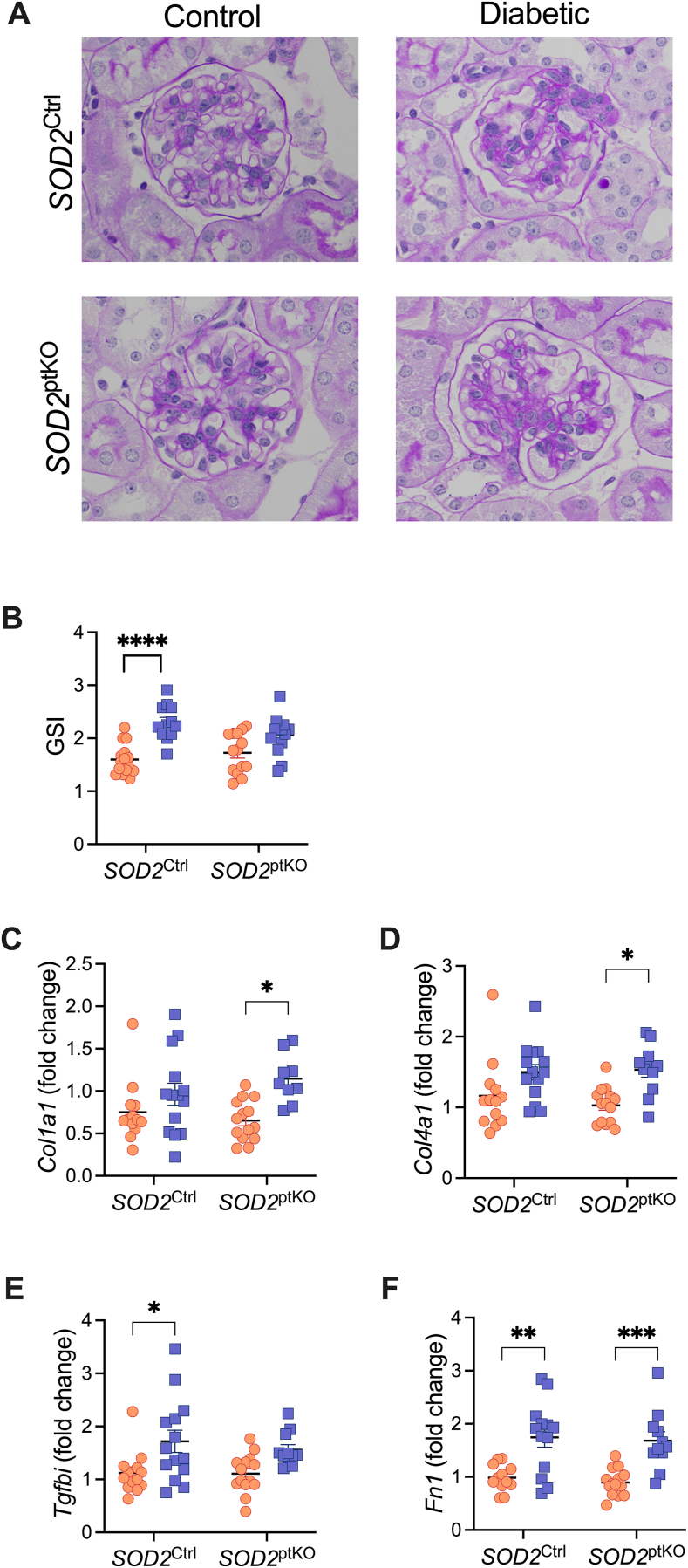
**Renal fibrosis.** A) Periodic acid-Schiff, B) Glomerulosclerosis index, n = 12 to 15 per group. Gene expression of C) Collagen 1, n = 10 to 14 per group. D) Collagen 4, n = 11 to 14 per group. E) Tgf-β, n = 11 to 14 per group, and F) Fibronectin, n = 11 to 14 per group. Dots and squares represent individual mice. Orange dots are non-diabetic mice. Blue squares are mice with diabetes (Akita). *P* values were determined by two-way ANOVA with Tukey's multiple comparison test. ∗*P* < 0.05, ∗∗*P* < 0.01, ∗∗∗*P* < 0.001, ∗∗∗∗*P* < 0.0001.

SOD2 deletion in the proximal tubules had little effect on damage to the glomerulus as shown by glomerulosclerosis (Fig. 3A–B), and did not appear to significantly impact mRNA expression of fibrotic markers in diabetic SOD2^ptKO^ when compared to diabetic SOD2^Ctrl^ mice (Fig. 3C–F). Of note, both collagen Type I and collagen Type IV mRNA expression were significantly increased in diabetic knockout mice (Fig. 3C–D), however this increase was not observed in diabetic control mice. Conversely, *Tgfbi* had increased expression in SOD2^Ctrl^ diabetic mice, but this increase was not observed in SOD2^ptKO^ mice (Fig. 3E). Fibronectin was significantly increased in the setting of diabetes in both genotypes (Fig. 3F).

### 3.3 Knockout of SOD2 in the proximal tubules worsens lipid peroxidation

**Fig. 4:**
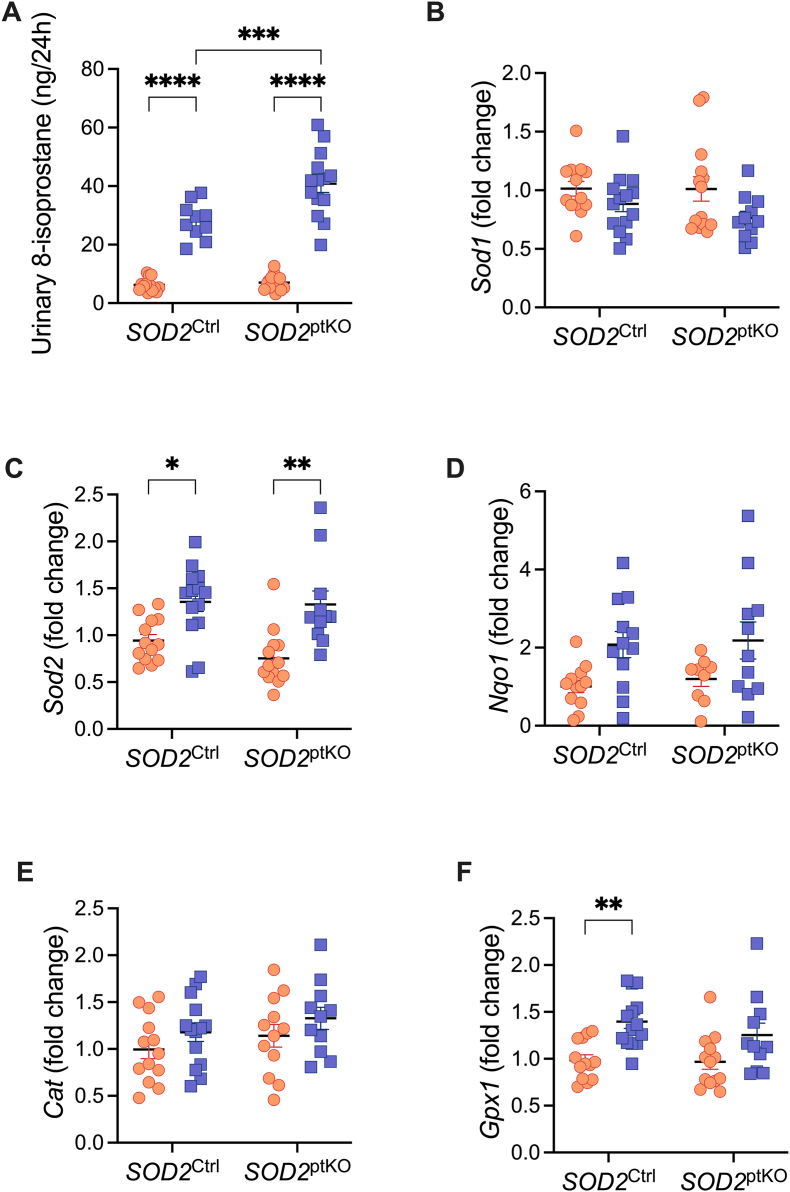
Biomarkers of oxidative stress A) Urinary 8-isoprostane, n = 10 to 14 per group. Gene expression of B) *Sod1*, n = 11 to 14 per group. C) *Sod2*, n = 11 to 14 per group. D) *Nqo1*, n = 9 to 12 per group. E) *Cat*, n = 11 to 14 per group. F) *Gpx1,* n = 11 to 14 per group. Dots and squares represent individual mice. Orange dots are non-diabetic mice. Blue squares are mice with diabetes (Akita). *P* values were determined by two-way ANOVA with Tukey's multiple comparison test. ∗*P* < 0.05, ∗∗*P* < 0.01, ∗∗∗*P* < 0.001, ∗∗∗∗*P* < 0.0001.

Urinary excretion of 15-isoprostane F_2t_ (8-isoprostane), a marker of oxidative stress, was significantly increased in the setting of diabetes in both SOD2^ptKO^ and SOD2^Ctrl^ genotypes (Fig. 4A), indicating an increased whole body oxidative stress status. Moreover, genetic ablation of SOD2 in the proximal tubules further exacerbated diabetes-induced oxidative stress (Fig. 4A). Copper–Zinc superoxide dismutase (SOD1) mRNA expression was not altered by diabetes and was not impacted by proximal tubular knockout of SOD2 (Fig. 4B). Manganese superoxide dismutase (SOD2) mRNA expression increased in diabetes, however expression was not altered by SOD2 knockout (Fig. 4C). Other mRNA markers of oxidative stress, *Nqo1* and *Cat* were unaltered by both diabetes and the knockout of SOD2 (Fig. 4D–E). Finally, mRNA expression of the enzyme glutathione peroxidase-1 (Gpx1), an important neutraliser of cellular ROS [19], was increased in the setting of diabetes in SOD2^Ctrl^ mice, however this was not observed in SOD2^ptKO^ mice (Fig. 4F).

In summary, the current study showed that diabetic mice with SOD2 proximal tubular knockout showed little difference in renal phenotype and function in comparison to diabetic SOD2^Ctrl^ littermates. Indicators of kidney damage and dysfunction including plasma cystatin C, KIM-1, and glomerulosclerosis, along with mRNA expression of fibrotic and oxidative stress gene markers were not significantly altered in diabetic SOD2^ptKO^ mice. Collectively, these results suggest that the role of proximal tubular SOD is more complex than previously anticipated. Notably, despite the absence of significant alterations in traditional markers of kidney injury, diabetic *SOD2*^ptKO^ mice exhibited an increase in urinary albumin, a hallmark of renal dysfunction. This observation highlights a potentially novel and yet-to-be-defined role for SOD2 in the kidney, warranting further investigation.

## CRediT authorship contribution statement

**Inez A. Trambas:** Writing – original draft, Formal analysis, Data curation. **Lilliana Bowen:** Writing – original draft, Visualization, Methodology, Investigation, Formal analysis, Data curation. **Vicki Thallas-Bonke:** Methodology, Investigation. **Matthew Snelson:** Investigation. **Karly C. Sourris:** Investigation. **Adrienne Laskowski:** Investigation. **Michel Tauc:** Resources. **Isabelle Rubera:** Resources. **Guoping Zheng:** Resources. **David C.H. Harris:** Resources. **Phillip Kantharidis:** Writing – review & editing, Validation, Methodology, Investigation. **Takahiko Shimizu:** Resources. **Mark E. Cooper:** Writing – review & editing, Resources, Funding acquisition. **Sih Min Tan:** Writing – review & editing, Validation, Supervision, Project administration, Investigation, Formal analysis, Data curation. **Melinda T. Coughlan:** Writing – review & editing, Writing – original draft, Visualization, Validation, Supervision, Software, Project administration, Methodology, Funding acquisition, Conceptualization.

## Funding

This work was supported by 10.13039/100022690JDRF (3-PDF-2014-106-A-N, 4-CDA-2018-613-*M*-B, 3-APF-2017-418-A-N). The funding bodies had no role in study design, collection, analysis and interpretation of data; in the writing of the paper; and in the decision to submit the article for publication.

## Declaration of competing interest

The authors declare that they have no known competing financial interests or personal relationships that could have appeared to influence the work reported in this paper.

## Data Availability

Data will be made available on request.
