# Supplementary material for: Proximal tubular deletion of superoxide dismutase-2 reveals disparate effects on kidney function in diabetes
Source: Redox Biol. 2025 Mar 18;82:103601. doi: 10.1016/j.redox.2025.103601 (PMC11979990; doi:10.1016/j.redox.2025.103601)
Supplement: Multimedia component 1 [file mmc1.docx]

**Supplementary Materials for Proximal tubular deletion of superoxide dismutase-2 reveals disparate effects on kidney function in diabetes**

Inez A. Trambas^1*^, Lilliana Bowen^1*^, Vicki Thallas-Bonke^1^, Matthew Snelson^1^, Karly C. Sourris^1^, Adrienne Laskowski^1^, Michel Tauc^2^, Isabelle Rubera^2^, Guoping Zheng^3^, David C. H. Harris^3^, Phillip Kantharidis^1^, Takahiko Shimizu^4^, Mark E. Cooper^1^, Sih Min Tan^1†^, Melinda T. Coughlan^1,5,6,*, †^

^1^Department of Diabetes, School of Translational Medicine, Monash University, Melbourne 3004, Victoria, Australia

^2^Laboratoire de Physiomédecine Moléculaire, Université Côte d'Azur, CNRS, LP2M, 7370, Nice Cedex 2, France

^3^Centre for Transplantation and Renal Research, Westmead Institute for Medical Research, University of Sydney, Sydney, NSW 2145, Australia.

^4^Department of Food and Reproductive Function Advanced Research, Juntendo University Graduate School of Medicine, Bunkyo-ku, Tokyo 113-8421, Japan

^5^Baker Heart and Diabetes Institute, Melbourne 3004, Victoria, Australia.

^6^Drug Discovery Biology, Monash Institute of Pharmaceutical Science, Monash University Parkville Campus, 381 Royal Parade, Parkville 3052, Victoria, Australia.

^*^equal first authorship

^†^equal senior authorship

**Corresponding Author:**

Melinda T. Coughlan

Glycation, Nutrition & Metabolism Laboratory

Department of Diabetes

School of Translational Medicine, Monash University

99 Commercial Road, Melbourne, 3004, Australia.

Email: melinda.coughlan@monash.edu

Twitter handle: @MelindaCoughlan

**This file includes:**

Figure S1

Figure S2


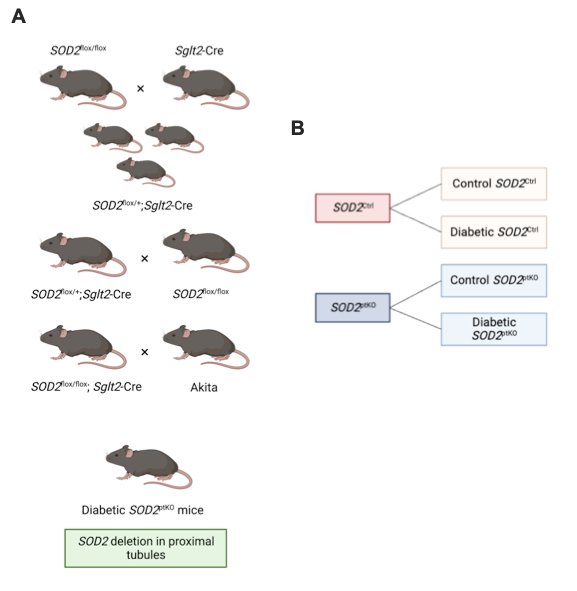


**Fig S1. Experimental design.**

A) Breeding of proximal tubule-specific deletion of SOD2. B) Animal genotypes.

**
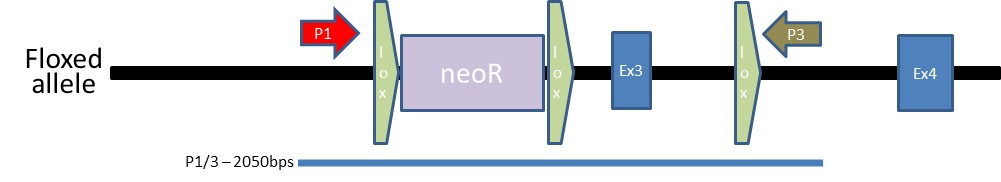
A**

**
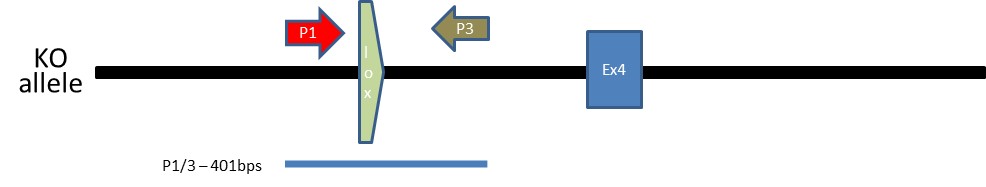
B**

**
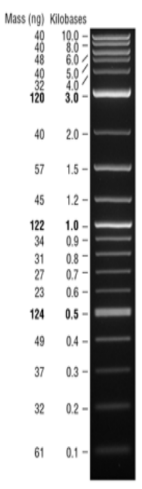
**

**C**


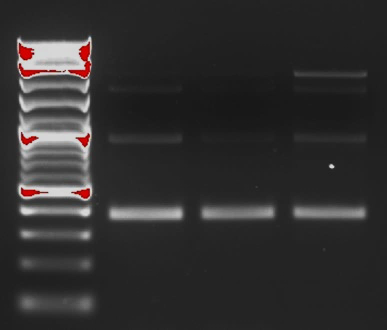


**Ladder Cortex Tubules Gloms**

**D**

***SOD2*^Ctrl^-Con *SOD2*^ptKO^-Con Negative Control**


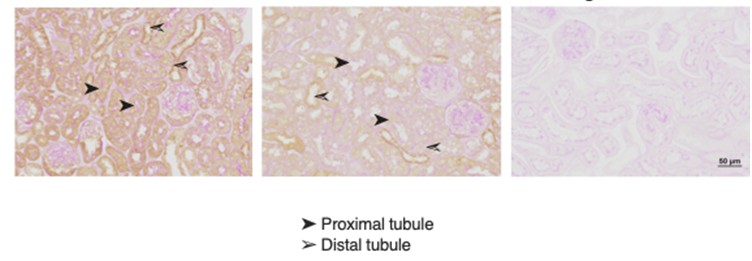


**Proximal Tubule**

**Distal Tubule**


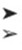


**Fig S2. Verification of the SOD2 knockout mouse model.** A) Primer map showing Floxed SOD2 allele and the loxP sites, and the 2050bp PCR product generated by P1/P3. B) Map showing the deletion of the SOD2 allele and the single remaining loxP site, and the 401bp PCR product generated by P1/P3. C) DNA gel showing the expected size band of 401bps from the KO allele in kidney tissue. D) SOD2 immunohistochemistry and periodic acid-Schiff stain to identify proximal tubule-specific *Sod2* knockout. Proximal tubules were identified by positive PAS-staining at the brush borders. X200 magnification, scale bar=50um. Closed arrows indicate proximal tubules and open arrows indicate distal tubules.
